# Predicting protein residue-residue contacts using random forests and deep networks

**DOI:** 10.1186/s12859-019-2627-6

**Published:** 2019-03-14

**Authors:** Joseph Luttrell, Tong Liu, Chaoyang Zhang, Zheng Wang

**Affiliations:** 1 School of Computing Sciences and Computer Engineering, University of Southern Mississippi, 118 College Drive, Hattiesburg, MS 39406 USA; 2 Department of Computer Science, University of Miami, 1365 Memorial Drive, Coral Gables, FL 33124 USA

**Keywords:** Residue-residue contact prediction, Protein, Random forest, Direct coupling analysis, Web server

## Abstract

**Background:**

The ability to predict which pairs of amino acid residues in a protein are in contact with each other offers many advantages for various areas of research that focus on proteins. For example, contact prediction can be used to reduce the computational complexity of predicting the structure of proteins and even to help identify functionally important regions of proteins. These predictions are becoming especially important given the relatively low number of experimentally determined protein structures compared to the amount of available protein sequence data.

**Results:**

Here we have developed and benchmarked a set of machine learning methods for performing residue-residue contact prediction, including random forests, direct-coupling analysis, support vector machines, and deep networks (stacked denoising autoencoders). These methods are able to predict contacting residue pairs given only the amino acid sequence of a protein. According to our own evaluations performed at a resolution of +/− two residues, the predictors we trained with the random forest algorithm were our top performing methods with average top 10 prediction accuracy scores of 85.13% (short range), 74.49% (medium range), and 54.49% (long range). Our ensemble models (stacked denoising autoencoders combined with support vector machines) were our best performing deep network predictors and achieved top 10 prediction accuracy scores of 75.51% (short range), 60.26% (medium range), and 43.85% (long range) using the same evaluation. These tests were blindly performed on targets from the CASP11 dataset; and the results suggested that our models achieved comparable performance to contact predictors developed by groups that participated in CASP11.

**Conclusions:**

Due to the challenging nature of contact prediction, it is beneficial to develop and benchmark a variety of different prediction methods. Our work has produced useful tools with a simple interface that can provide contact predictions to users without requiring a lengthy installation process. In addition to this, we have released our C++ implementation of the direct-coupling analysis method as a standalone software package. Both this tool and our RFcon web server are freely available to the public at http://dna.cs.miami.edu/RFcon/.

**Electronic supplementary material:**

The online version of this article (10.1186/s12859-019-2627-6) contains supplementary material, which is available to authorized users.

## Background

The field of residue-residue contact prediction attempts to solve the problem of predicting which amino acid residues in the structure of a protein are “in contact”. Typically, residues are defined to be in contact when the distance between their β-carbon atoms (or α-carbon for the amino acid glycine) is smaller than 8 Å [1]. The ability to make predictions about which residues within a protein fall within these parameters can assist researchers by providing information about the native structure and other physical properties of that protein before they expend valuable resources on physical experiments [2]. This is especially true since evidence suggests that intra-molecular interactions among residues play an important role in determining the overall stability of a protein’s native structure [3]. Therefore, these predictions can be useful in computational drug design, identification of functional sites on proteins, and many other areas of research that study the properties of proteins [4, 5].

Regardless of the intended applications, the primary goal of contact prediction research is typically to produce predictions that correctly label a pair of residues in a protein as “in contact” or “not in contact”. Therefore, many of the challenges in this field can be naturally approached as classification problems [6]. In general, contact prediction methods are organized as either sequence-based or template-based. Sequence-based contact prediction research typically utilizes machine learning methods and explores a wide variety of techniques such as support vector machines (SVMs) [7, 8], neural networks [9], random forests (RF) [10, 11], and convolutional neural networks (CNNs) [12, 13]. While these methods vary in the technical details of their approach, they often share the common goal of discovering patterns in protein data that appear when residue pairs are observed to be in contact.

Zhang et al. developed a hybrid SVM contact predictor that incorporated mixed integer linear programming (MILP) in cases where the SVM model was not able to make a prediction below a certain confidence threshold [7]. Their approach is specifically tailored to deal with the challenges of predicting contacts within transmembrane (TM) proteins. Cheng and Baldi focused on increasing the performance of their SVMs by improving their training data with feature selection techniques [8]. Eickholt and Cheng chose a different approach based on deep networks and boosting [9]. Their method used restricted Boltzmann machines (RBMs) combined to form deep networks (DNs) and was improved by using boosting to optimize the weights of the models during training. Li et al. used RF models and were able to improve the accuracy of their previous methods by focusing on feature design and feature selection [10]. Recently, convolutional neural networks have been gaining popularity in contact prediction studies. Wang et al. combined evolutionary coupling and sequence conservation information to train very deep networks with 60–70 convolutional layers [13]. Adhikari et al. focused on using convolutional neural networks to directly predict contact maps by designing input volumes based on two-dimensional channels of features [12]. Their predictor, DNCON2, was able to achieve mean precision scores of 35, 50, and 53.4% in the CASP10, 11, and 12 experiments, respectively. These predicted contact maps were generated by a two level system with the first layer containing five CNNs that predict preliminary contact probabilities at different distance thresholds and the second level containing one CNN that combines the input feature volume with the 2D contact probabilities that were predicted in the first level.

Template-based contact prediction methods typically focus less on training from general data and instead choose to make more informed predictions by making decisions based on real-world contact information from the large amount of experimentally obtained protein structures that are available. These methods typically rely on homology or threading to identify similarities between previously known protein structures (templates) and a query protein. The residue interaction data obtained from those templates can then be used to produce contact predictions that may be more biologically relevant than predictions from more general methods [14, 15]. Some template-based methods go beyond simply searching for contacts in existing template structures and incorporate machine learning techniques into their predictions. For example, Wu and Zhang were able to combine SVMs with data from multiple threading methods into a contact predictor which they named SVM-LOMETS [14].

The methods we present here are sequence-based and incorporate various types of data and machine learning techniques. While our highest performing models were produced by the random forest (RF) algorithm, we also utilized methods based on support vector machines (SVM), stacked denoising autoencoders (SDA), and direct coupling analysis (DCA). Here we compare them and evaluate their performance against the results of our own evaluation of contact predictors produced by other research groups in recent years. Our final RF and SDA methods are provided for use as part of a publically accessible web server [16].

## Results

### Evaluation metrics

Accuracy (acc) and coverage (cov) formed the basis of our evaluation during training and testing. Here, *TP* is the number of residue pairs that were correctly predicted to be in contact, *FP* is the number of residue pairs that were incorrectly predicted to be in contact, and *nativePositives* is the total number of residue pairs that were in contact in the native structure of the protein being evaluated.

1 $$ acc=\frac{TP}{TP+ FP} $$

2 $$ \operatorname{cov}=\frac{TP}{nativePostives} $$

### Blind test performance evaluation

Tables 1, 2, 3, 4, 5, 6, 7, 8, 9 and 10 depict the performance results of our blind test of our own contact prediction methods (bold method names in these tables) and a selection of the contact predictors which participated in CASP11. Tables 11, 12, 13, 14, 15 and 16 describe the same type of results but from the models described in the previously mentioned Additional Models section. These tests were performed using the target proteins from the CASP11 experiment and ensure that none of the evaluated prediction methods were trained on proteins that exist in this testing set. Also, as described in our methods section, our training data was filtered to remove proteins with sequences that were too similar to the group of test proteins. The predictors listed here are not ranked in any particular order, and the values given are averages of accuracy and coverage calculations using the procedures defined in our methods section.

**Table 1:** Short range accuracy evaluation 1. Average short range accuracy values at *δ* = 2 resolution for the top 10, top L/10, and top L/5 contact predictions from various predictors (with our methods listed in bold font) evaluated on CASP11 targets and sorted by Top 10 accuracy scores

Method	Top 10	L/10	L/5
CONSIP2 (94)	91.29%	87.23%	83.29%
RaptorX-Contact (94)	89.89%	87.02%	83.17%
MULTICOM-NOVEL (94)	85.93%	84.16%	82.73%
**rf_full (78)**	85.13%	82.07%	77.37%
**rf_select (78)**	80.38%	77.63%	73.83%
PLCT (91)	75.70%	76.46%	76.50%
**sda_Ensemble (78)**	75.51%	72.53%	71.39%
**svm (78)**	73.08%	72.61%	72.57%
**sda_balanced (78)**	71.79%	68.89%	66.28%
**sda_unbalanced (78)**	71.77%	71.50%	71.50%
IASL-COPE (91)	54.67%	53.23%	53.22%
Pcons-net (87)	49.64%	47.98%	47.76%
raghavagps-paaint (76)	46.45%	47.18%	47.21%
**DCA_cpp(88)**	38.54%	36.71%	34.57%
FoDTcm (32)	31.25%	34.01%	34.95%

**Table 2:** Medium range accuracy evaluation 1. Average medium range accuracy values at *δ* = 2 resolution for the top 10, top L/10, and top L/5 contact predictions from various predictors (with our methods listed in bold font) evaluated on CASP11 targets and sorted by Top 10 accuracy scores

Method	Top 10	L/10	L/5
RaptorX-Contact (94)	84.68%	80.57%	77.90%
CONSIP2 (94)	84.50%	82.18%	79.28%
MLiD (94)	77.34%	74.82%	70.92%
**rf_full (78)**	74.49%	73.48%	69.91%
**rf_select (78)**	65.51%	63.28%	61.47%
**svm (78)**	62.18%	60.60%	58.76%
**sda_Ensemble (78)**	60.26%	59.59%	57.15%
MULTICOM-NOVEL (94)	58.41%	57.59%	57.22%
IASL-COPE (91)	55.97%	52.63%	52.54%
**sda_balanced (78)**	54.87%	53.76%	51.96%
PLCT (91)	54.82%	55.65%	55.91%
Pcons-net (87)	53.22%	52.18%	51.85%
**DCA_cpp (88)**	50.85%	44.89%	40.65%
**sda_unbalanced (78)**	44.93%	46.50%	46.69%
raghavagps-paaint (76)	43.29%	44.04%	43.84%
FoDTcm (32)	25.00%	30.79%	30.60%

**Table 3:** Long range accuracy evaluation 1. Average long range accuracy values at *δ* = 2 resolution for the top 10, top L/10, and top L/5 contact predictions from various predictors (with our methods listed in bold font) evaluated on CASP11 targets and sorted by Top 10 accuracy scores

Method	Top 10	L/10	L/5
CONSIP2 (94)	71.71%	70.18%	67.30%
RaptorX-Contact (94)	61.60%	56.80%	55.63%
**rf_full (78)**	54.49%	52.98%	52.09%
MLiD (94)	53.51%	49.98%	46.99%
**svm (78)**	52.44%	48.75%	46.25%
**rf_select (78)**	50.77%	50.05%	48.66%
**DCA _cpp(88)**	47.07%	44.10%	40.82%
**sda_Ensemble (78)**	43.85%	42.65%	42.16%
**sda_unbalanced (78)**	37.91%	38.81%	37.78%
IASL-COPE (91)	37.58%	34.87%	33.70%
**sda_balanced (78)**	35.51%	33.90%	34.19%
MULTICOM-NOVEL (94)	27.97%	27.25%	27.12%
raghavagps-paaint (76)	26.50%	26.37%	26.68%
Pcons-net (87)	17.72%	18.41%	18.93%
FoDTcm (32)	17.71%	17.45%	18.10%
PLCT (91)	16.87%	17.24%	17.08%

**Table 4:** Short range accuracy evaluation 2. Average short range accuracy values at *δ* = 1 resolution for the top 10, top L/10, and top L/5 contact predictions from various predictors (with our methods listed in bold font) evaluated on CASP11 targets and sorted by Top 10 accuracy scores

Method	Top 10	L/10	L/5
CONSIP2 (94)	88.01%	82.35%	76.21%
RaptorX-Contact (94)	80.96%	78.93%	73.89%
**rf_full (78)**	78.59%	73.15%	66.05%
MULTICOM-NOVEL (94)	78.52%	75.60%	74.03%
**rf_select (78)**	74.74%	69.23%	63.14%
PLCT (91)	71.17%	72.42%	72.54%
**sda_Ensemble (78)**	68.21%	63.92%	59.56%
**sda_unbalanced (78)**	62.52%	61.87%	61.90%
**sda_balanced (78)**	61.67%	57.52%	53.51%
**svm (78)**	59.23%	57.88%	56.81%
IASL-COPE (91)	47.46%	45.43%	45.36%
Pcons-net (87)	45.83%	44.11%	43.88%
raghavagps-paaint (76)	34.47%	35.89%	35.43%
**DCA (88)**	31.46%	27.34%	25.51%
FoDTcm (32)	19.06%	21.50%	22.34%
MLiD (94)	N/A	N/A	N/A

**Table 5:** Medium range accuracy evaluation 2. Average medium range accuracy values at *δ* = 1 resolution for the top 10, top L/10, and top L/5 contact predictions from various predictors (with our methods listed in bold font) evaluated on CASP11 targets and sorted by Top 10 accuracy scores

Method	Top 10	L/10	L/5
CONSIP2 (94)	80.74%	77.50%	73.01%
RaptorX-Contact (94)	77.77%	73.34%	68.28%
MLiD (94)	66.49%	63.34%	58.86%
**rf_full (78)**	61.15%	60.06%	56.63%
**rf_select (78)**	56.41%	53.23%	49.81%
MULTICOM-NOVEL (94)	54.84%	53.89%	52.80%
PLCT (91)	51.93%	53.02%	53.36%
Pcons-net (87)	49.87%	48.60%	48.02%
**sda_Ensemble (78)**	48.08%	46.78%	43.54%
**svm (78)**	47.18%	43.87%	42.41%
IASL-COPE (91)	45.78%	42.75%	42.55%
**DCA (88)**	42.32%	36.06%	31.16%
**sda_balanced (78)**	40.90%	39.06%	37.39%
**sda_unbalanced (78)**	33.08%	33.39%	34.02%
raghavagps-paaint (76)	32.63%	32.22%	32.48%
FoDTcm (32)	11.25%	17.87%	18.87%

**Table 6:** Long range accuracy evaluation 2. Average long range accuracy values at *δ* = 1 resolution for the top 10, top L/10, and top L/5 contact predictions from various predictors (with our methods listed in bold font) evaluated on CASP11 targets and sorted by Top 10 accuracy scores

Method	Top 10	L/10	L/5
CONSIP2 (94)	63.86%	62.60%	58.92%
RaptorX-Contact (94)	52.13%	47.50%	45.58%
**rf_full (78)**	45.64%	43.45%	40.88%
MLiD (94)	42.77%	39.65%	37.26%
**rf_select (78)**	42.18%	39.99%	36.88%
**svm (78)**	42.05%	36.87%	34.20%
**DCA (88)**	39.76%	36.20%	33.73%
**sda_Ensemble (78)**	32.82%	31.02%	29.85%
IASL-COPE (91)	32.07%	28.93%	27.30%
**sda_unbalanced (78)**	29.17%	27.67%	26.52%
MULTICOM-NOVEL (94)	25.73%	25.21%	25.11%
**sda_balanced (78)**	21.60%	20.91%	21.60%
raghavagps-paaint (76)	17.63%	18.13%	18.41%
PLCT (91)	15.67%	16.61%	16.46%
Pcons-net (87)	15.29%	15.85%	16.27%
FoDTcm (32)	8.55%	8.89%	8.73%

**Table 7:** Short range accuracy evaluation 3. Average short range accuracy values at *δ* = 0 resolution for the top 10, top L/10, and top L/5 contact predictions from various predictors (with our methods listed in bold font) evaluated on CASP11 targets and sorted by Top 10 accuracy scores

Method	Top 10	L/10	L/5
CONSIP2 (94)	76.18%	66.18%	55.85%
MULTICOM-NOVEL (94)	60.58%	53.84%	50.71%
PLCT (91)	54.94%	57.13%	57.47%
RaptorX-Contact (94)	54.89%	50.02%	43.04%
**rf_full (78)**	52.18%	42.91%	35.28%
**rf_select (78)**	46.41%	38.68%	31.11%
**sda_unbalanced (78)**	39.77%	38.67%	38.80%
**sda_Ensemble (78)**	39.49%	33.57%	28.57%
Pcons-net (87)	37.32%	34.20%	33.66%
**sda_balanced (78)**	35.38%	30.67%	25.13%
IASL-COPE (91)	33.01%	30.40%	30.36%
**svm (78)**	30.90%	28.40%	24.64%
**DCA_cpp (88)**	18.78%	14.74%	12.85%
raghavagps-paaint (76)	15.92%	15.46%	15.14%
FoDTcm (32)	5.00%	7.29%	8.05%
MLiD (94)	N/A	N/A	N/A

**Table 8:** Medium range accuracy evaluation 3. Average medium range accuracy values at *δ* = 0 resolution for the top 10, top L/10, and top L/5 contact predictions from various predictors (with our methods listed in bold font) evaluated on CASP11 targets and sorted by Top 10 accuracy scores

Method	Top 10	L/10	L/5
CONSIP2 (94)	70.76%	63.23%	54.96%
RaptorX-Contact (94)	51.91%	46.01%	39.47%
Pcons-net (87)	43.51%	40.64%	39.66%
MLiD (94)	41.01%	37.69%	32.81%
PLCT (91)	39.02%	40.10%	40.85%
MULTICOM-NOVEL (94)	38.75%	36.89%	34.79%
**rf_full (78)**	33.08%	29.68%	26.09%
IASL-COPE (91)	32.14%	29.43%	29.02%
**rf_select (78)**	31.28%	26.68%	22.65%
**DCA_cpp (88)**	30.98%	25.07%	19.91%
**sda_Ensemble (78)**	23.72%	23.01%	19.64%
**svm (78)**	18.33%	17.03%	16.64%
**sda_unbalanced (78)**	16.99%	15.51%	14.25%
**sda_balanced (78)**	15.90%	16.90%	16.05%
raghavagps-paaint (76)	13.42%	13.16%	13.69%
FoDTcm (32)	1.56%	4.18%	5.22%

**Table 9:** Long range accuracy evaluation 3. Average long range accuracy values at *δ* = 0 resolution for the top 10, top L/10, and top L/5 contact predictions from various predictors (with our methods listed in bold font) evaluated on CASP11 targets and sorted by Top 10 accuracy scores

Method	Top 10	L/10	L/5
CONSIP2 (94)	36.73%	38.14%	35.97%
RaptorX-Contact (94)	32.45%	29.04%	26.58%
**DCA_cpp (88)**	32.07%	28.15%	24.79%
MLiD (94)	27.23%	23.70%	20.59%
**rf_full (78)**	25.00%	21.80%	18.98%
IASL-COPE (91)	24.80%	20.70%	19.44%
**rf_select (78)**	22.82%	19.94%	18.41%
MULTICOM-NOVEL (94)	18.44%	17.42%	17.43%
**svm (78)**	17.69%	14.88%	13.36%
**sda_Ensemble (78)**	15.13%	13.50%	12.56%
**sda_unbalanced (78)**	14.07%	11.87%	11.45%
Pcons-net (87)	12.53%	12.99%	12.82%
PLCT (91)	12.01%	12.75%	12.69%
**sda_balanced (78)**	9.04%	7.76%	7.50%
raghavagps-paaint (76)	6.05%	7.27%	7.16%
FoDTcm (32)	1.65%	1.46%	1.63%

**Table 10:** Coverage evaluation. Average short, medium (Med), and long range coverage values at *δ* = 0 resolution for the top L/10 contact predictions from various predictors (with our methods listed in bold font) evaluated on CASP11 targets and sorted by short range scores

Method	Short	Med	Long
CONSIP2 (94)	22.54%	15.66%	4.07%
RaptorX-Contact (94)	17.27%	12.03%	2.21%
**rf_full (78)**	14.45%	7.62%	1.68%
MULTICOM-NOVEL (94)	13.37%	5.66%	0.44%
**rf_select (78)**	13.18%	6.75%	1.51%
**sda_Ensemble (78)**	11.09%	5.81%	1.05%
**sda_balanced (78)**	10.20%	4.47%	0.58%
**svm (78)**	9.53%	4.33%	1.18%
PLCT (91)	7.25%	3.59%	0.36%
Pcons-net (87)	7.00%	7.50%	2.73%
raghavagps-paaint (76)	5.05%	3.60%	0.65%
**DCA (88)**	5.02%	6.74%	2.20%
IASL-COPE (91)	4.41%	3.93%	1.24%
**sda_unbalanced (78)**	3.87%	3.74%	0.91%
FoDTcm (32)	2.06%	0.98%	0.15%
MLiD (94)	N/A	9.69%	1.81%

**Table 11:** Short range accuracy evaluation 4 with 50% less training data. Average short range accuracy values at *δ* = 2 resolution for the top 10, top L/10, and top L/5 contact predictions from various predictors (with our methods listed in bold font) evaluated on CASP11 targets and sorted by Top 10 accuracy scores. These models use half of the number of examples (data points) for training

Method	Top 10	L/10	L/5
CONSIP2 (94)	91.29%	87.23%	83.29%
RaptorX-Contact (94)	89.89%	87.02%	83.17%
MULTICOM-NOVEL (94)	85.93%	84.16%	82.73%
**rf_full (78)**	85.26%	81.77%	77.30%
**rf_select (78)**	81.03%	77.78%	74.18%
PLCT (91)	75.70%	76.46%	76.50%
**svm (78)**	74.10%	72.93%	71.99%
IASL-COPE (91)	54.67%	53.23%	53.22%
Pcons-net (87)	49.64%	47.98%	47.76%
raghavagps-paaint (76)	46.45%	47.18%	47.21%
**DCA_cpp(88)**	38.54%	36.71%	34.57%
FoDTcm (32)	31.25%	34.01%	34.95%

**Table 12:** Medium range accuracy evaluation 4 with 50% less training data. Average medium range accuracy values at *δ* = 2 resolution for the top 10, top L/10, and top L/5 contact predictions from various predictors (with our methods listed in bold font) evaluated on CASP11 targets and sorted by Top 10 accuracy scores. These models use half of the number of examples (data points) for training

Method	Top 10	L/10	L/5
RaptorX-Contact (94)	84.68%	80.57%	77.90%
CONSIP2 (94)	84.50%	82.18%	79.28%
MLiD (94)	77.34%	74.82%	70.92%
**rf_full (78)**	73.59%	71.59%	69.25%
**rf_select (78)**	65.26%	62.98%	60.57%
**svm (78)**	61.67%	59.77%	58.37%
MULTICOM-NOVEL (94)	58.41%	57.59%	57.22%
IASL-COPE (91)	55.97%	52.63%	52.54%
PLCT (91)	54.82%	55.65%	55.91%
Pcons-net (87)	53.22%	52.18%	51.85%
**DCA_cpp (88)**	50.85%	44.89%	40.65%
raghavagps-paaint (76)	43.29%	44.04%	43.84%
FoDTcm (32)	25.00%	30.79%	30.60%

**Table 13:** Long range accuracy evaluation 4 with 50% less training data. Average long range accuracy values at *δ* = 2 resolution for the top 10, top L/10, and top L/5 contact predictions from various predictors (with our methods listed in bold font) evaluated on CASP11 targets and sorted by Top 10 accuracy scores. These models use half of the number of examples (data points) for training

Method	Top 10	L/10	L/5
CONSIP2 (94)	71.71%	70.18%	67.30%
RaptorX-Contact (94)	61.60%	56.80%	55.63%
MLiD (94)	53.51%	49.98%	46.99%
**rf_full (78)**	52.31%	52.50%	51.59%
**rf_select (78)**	50.64%	49.73%	47.36%
**svm (78)**	50.00%	47.24%	46.26%
**DCA _cpp(88)**	47.07%	44.10%	40.82%
IASL-COPE (91)	37.58%	34.87%	33.70%
MULTICOM-NOVEL (94)	27.97%	27.25%	27.12%
raghavagps-paaint (76)	26.50%	26.37%	26.68%
Pcons-net (87)	17.72%	18.41%	18.93%
FoDTcm (32)	17.71%	17.45%	18.10%
PLCT (91)	16.87%	17.24%	17.08%

**Table 14:** Short range accuracy evaluation 5 with alternative feature selection. Average short range accuracy values at *δ* = 2 resolution for the top 10, top L/10, and top L/5 contact predictions from various predictors (with our methods listed in bold font) evaluated on CASP11 targets and sorted by Top 10 accuracy scores. These models were trained with features selected according to the mean decrease accuracy metric instead of mean decrease Gini

Method	Top 10	L/10	L/5
CONSIP2 (94)	91.29%	87.23%	83.29%
RaptorX-Contact (94)	89.89%	87.02%	83.17%
MULTICOM-NOVEL (94)	85.93%	84.16%	82.73%
**rf_select (78)**	81.54%	79.24%	74.52%
**svm (78)**	76.79%	75.82%	74.47%
PLCT (91)	75.70%	76.46%	76.50%
**sda_balanced (78)**	74.87%	75.14%	73.81%
**sda_unbalanced (78)**	74.52%	74.39%	74.33%
IASL-COPE (91)	54.67%	53.23%	53.22%
Pcons-net (87)	49.64%	47.98%	47.76%
raghavagps-paaint (76)	46.45%	47.18%	47.21%
**DCA_cpp(88)**	38.54%	36.71%	34.57%
FoDTcm (32)	31.25%	34.01%	34.95%

**Table 15:** Medium range accuracy evaluation 5 with alternative feature selection. Average medium range accuracy values at *δ* = 2 resolution for the top 10, top L/10, and top L/5 contact predictions from various predictors (with our methods listed in bold font) evaluated on CASP11 targets and sorted by Top 10 accuracy scores. These models were trained with features selected according to the mean decrease accuracy metric instead of mean decrease Gini

Method	Top 10	L/10	L/5
RaptorX-Contact (94)	84.68%	80.57%	77.90%
CONSIP2 (94)	84.50%	82.18%	79.28%
MLiD (94)	77.34%	74.82%	70.92%
**rf_select (78)**	66.03%	62.45%	60.41%
MULTICOM-NOVEL (94)	58.41%	57.59%	57.22%
**svm (78)**	58.08%	58.77%	57.26%
IASL-COPE (91)	55.97%	52.63%	52.54%
**sda_balanced (78)**	55.14%	52.85%	51.50%
PLCT (91)	54.82%	55.65%	55.91%
Pcons-net (87)	53.22%	52.18%	51.85%
**DCA_cpp (88)**	50.85%	44.89%	40.65%
raghavagps-paaint (76)	43.29%	44.04%	43.84%
**sda_unbalanced (78)**	38.59%	43.43%	45.09%
FoDTcm (32)	25.00%	30.79%	30.60%

**Table 16:** Long range accuracy evaluation 5 with alternative feature selection. Average long range accuracy values at *δ* = 2 resolution for the top 10, top L/10, and top L/5 contact predictions from various predictors (with our methods listed in bold font) evaluated on CASP11 targets and sorted by Top 10 accuracy scores. These models were trained with features selected according to the mean decrease accuracy metric instead of mean decrease Gini

Method	Top 10	L/10	L/5
CONSIP2 (94)	71.71%	70.18%	67.30%
RaptorX-Contact (94)	61.60%	56.80%	55.63%
**svm (78)**	56.67%	54.62%	50.60%
MLiD (94)	53.51%	49.98%	46.99%
**rf_select (78)**	52.44%	52.30%	51.09%
**DCA _cpp(88)**	47.07%	44.10%	40.82%
**sda_balanced (78)**	46.79%	46.16%	43.76%
**sda_unbalanced (78)**	40.14%	38.25%	37.12%
IASL-COPE (91)	37.58%	34.87%	33.70%
MULTICOM-NOVEL (94)	27.97%	27.25%	27.12%
raghavagps-paaint (76)	26.50%	26.37%	26.68%
Pcons-net (87)	17.72%	18.41%	18.93%
FoDTcm (32)	17.71%	17.45%	18.10%
PLCT (91)	16.87%	17.24%	17.08%

We have listed the number of targets that were available for evaluation next to the name of each predictor since some groups did not produce predictions for all of the possible target proteins. The accuracy tables are divided into sequence separation categories where contact predictions are organized and evaluated by “short range” (sep6) in Table 1, Table 4, and Table 7; “medium range” (sep12) in Table 2, Table 5, and Table 8; and “long range” (sep24) in Table 3, Table 6, and Table 9. For the tables corresponding to the models described in the Additional Models section, the same format was used where “short range” (sep6) results can be found in Table 11 and Table 14, “medium range” (sep12) in Table 12 and Table 15, and “long range” (sep24) in Table 13 and Table 16. The process for separating predictions into these three categories is the same as the one described in the methods section for labeling our training examples. Each numerical column in the tables represents evaluations done at the different list evaluation sizes of “Top 10”, “L/10”, and “L/5”. Here, L represents the sequence length of each target protein.

For example, a list size of L/10 indicates that a number of the top predictions from each predictor equal to the number of the protein’s length divided by 10 are included for each target protein. We define the “top” predictions to be the first predictions present in the prediction file for each predictor. Here we define “Top 10” as the first 10 predictions provided by a predictor for any given target, and every table is sorted by the value of this score. Also, each of our tables is presented at various prediction resolution levels as indicated by the value of the *δ* parameter listed in the table legends. This value indicates the size of the contact “neighborhood” as it is described by Eickholt et al. [17]. Since coverage values begin to lose meaning at smaller evaluation list sizes, we have only included a coverage table for the L/10 prediction category at the resolution of *δ* = 0 that shows all three contact ranges (Table 10).

The *δ* parameter allows for contact predictions to be counted as correct even if they are off by a number of residues in the sequence equal to or less than the given value. The number of pairs in this “neighborhood” of acceptable predictions is defined by the following formula where *δ* is the resolution value and *n* is the resulting number of residue pairs that will be counted as correct (including the true pair in the experimentally determined structure).

3 $$ n={\left(2\delta +1\right)}^2 $$

For example, a contact prediction of the residue pair (5, 20) with *δ* = 0 would be counted as correct only if the 5th and 20th residues in the sequence were actually in contact in the experimentally determined protein structure. However, changing the resolution parameter to *δ* = 1 will add eight additional contact pairs to the list of “correct” contacts. In general, larger values of *δ* will allow for an increase in the perceived accuracy of a contact predictor as long as it is still able to predict contacting pairs that are within a reasonable distance of truly contacting pairs in the experimentally determined structure.

**Fig. 1:**
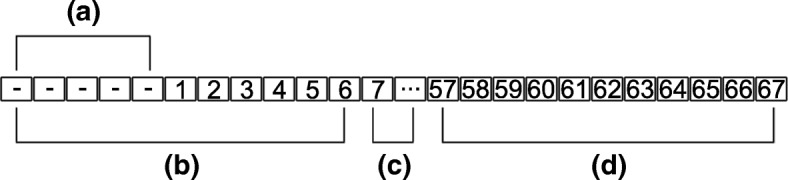
Selected visualizations of long-range contact predictions from our “rf_full” model (*δ* = 0). **a** The 3D structure of CASP target T0778 showing a selection of four correctly predicted residue pairs that are in contact (30–61, 33–57, 51–77, 55–91). **b** A contact map for T0778 showing its true contacts (blue points in the lower right triangle), the top L/2 predicted contacts from the “rf_full” model (red points in the upper left triangle), and the locations of the interactions between the helices (H1-H4). **c** The 3D structure of T0778 showing four helices (H1-H4) which contain the correctly predicted residue pairs. **d** The 3D structure of CASP target T0798 showing a selection of four correctly predicted residue pairs (7–81, 8–58, 80–113, 114–144). **e** A contact map for T0798 showing its true contacts (blue points in the lower right triangle), and the top L/2 predicted contacts from the rf_full model (red points in the upper left triangle). **f** The 3D structure of T0798 showing five strands (S1-S5) which contain correctly predicted residue pairs. Please note that the coloration in sections (a) and (d) is only used to show pairs of residues and is not intended to compare contacts between the two structures. The coloration in sections (c) and (f) indicates each residue’s numerical position in the sequence of the structure. This coloring starts at the n-terminal with dark blue and continues to light blue, green, yellow, orange, and finally red at the c-terminal

Figure 1 depicts a selection of the long range predictions from our “rf_full” model on two CASP11 targets and illustrates how our predictions can provide information about contacting residues that are separated by considerable sequence distance. For example, Fig. 1d includes a selection of our correctly predicted contacting residue pairs that reveals long range contact activity between five β-strands (S1, S2, S3, S4, and S5 labeled in Fig. 1f) within CASP11 target T0798. The correctly predicted residue pair with the longest sequence distance in this example (residue 7 and residue 81) is separated by 74 residues in the sequence of T0798. Figure 1e is a contact map that shows the distribution of our “rf_full” model’s long range predictions for T0798 (red points in the upper left corner) and the true contacting residue pairs for T0798 (blue points in the lower right corner). We have also labeled the location of the interacting β-strands (S1, S2, S3, S4, S5) in the upper left corner of Fig. 1e. The visualization of the data used to create our contact maps in Fig. 1b and e was done with the python library ContactVis [18]. The visualization of the structures of T0798 and T0778 that were used in Fig. 1a, c, d, and f was done with UCSF Chimera [19].

## Discussion

We trained and tested a collection of RF, SDA, and SVM models that are able to predict residue-residue contact in a protein given only that protein’s amino acid sequence as input. We also reported evaluation results for our DCA method (“DCA_cpp”) that was trained prior to this study. Our final models were selected by adjusting training parameters to reach higher values of accuracy (acc) and coverage (cov) during our training process. We chose a selection of the highest performing models from each method to participate in a blind test (testing the trained models on unseen protein targets) with a selection of predictors from CASP11. Our methods were able to provide reasonably competitive predictions in evaluation conditions with higher values of *δ* and a small enough list evaluation size.

According to our evaluation, our “rf_full” models produced the best results within our own group of models on average. This was especially true for evaluations at larger values of *δ* where the short range “rf_full” model was able to achieve an average accuracy of 85.13% at *δ* = 2 (Table 1) Even though these models show lower accuracy without the benefit of being evaluated at larger values of *δ*, they seem to be identifying many of the correct regions of contacting residues since their accuracy improves significantly upon increasing *δ*. Of course, this generally causes an increase in the perceived performance of other predictors as well. We believe that presenting accuracy scores with a resolution of *δ* = 1 or *δ* = 2 still allows for a realistic interpretation of the usability of the contact predictions.

For example, we list the long range accuracy of our “rf_full” model on the CASP11 dataset as 21.80% on the list size of L/10 with a resolution of *δ* = 0 (Table 9). With the same scoring parameters, we gave the generally high performing MLiD predictor an accuracy score of only 23.70% (Table 9). It is possible that many users of these prediction programs would interpret these results to be almost unusable for obtaining useful clues about which regions of their protein may contain some contacting residues. However, by simply increasing the size of the contact neighborhood by setting *δ* = 2 and leaving the list size at L/10, we see that the reported accuracies increase to 52.98% for our “rf_full” model and 49.98% for MLiD (Table 3). Of course, due to the relatively higher difficulty of long range contact prediction, many of the tables for short range and medium range show results with even higher accuracy values.

## Conclusions

Being able to use contact predictions to estimate the locations of interacting regions within a protein is beneficial to many areas of research involving proteins and protein structure. For example, having even a small amount of accurate contact predictions can help to reduce the computational complexity of predicting a protein’s structure by reducing the size of the search space of possible conformations. We have used different methods to perform contact prediction and have provided a comparison between deep learning (sda) and more traditional methods using our own evaluation. The results of our evaluation on our own models have shown that our rf_full models are capable of providing correct contact predictions in several key areas of interaction within proteins with high accuracy when compared to our other methods. Residue-residue contact prediction is a steadily improving field with many new techniques and theories being tested and developed each year. Therefore, future work for this project will likely involve improving the accuracy of our contact predictions by exploring different prediction methods and types of data.

## Methods

### Dataset sources

Our initial training dataset was composed of X-ray determined protein structures obtained by an advanced search of the Protein Data Bank [20] that filtered the returned results by 30% sequence similarity and an x-ray resolution of 0-2 Å. The choice of filtering for only X-ray determined protein structures at this resolution was made to minimize any variation between structures in our data that may be caused by using different experimental methods with widely varying resolution levels. Filtering the sequences by 30% sequence similarity prevented duplicates from appearing in the data and helped keep the training data more diverse. The next stage of filtering removed structures with more than 20% disordered residue content (more than 20% of the residues in the sequence were not represented in the experimentally determined list of atomic coordinates) and structures with sequences that were not within the range of 30 to 300 residues in length. These two steps were mainly performed to improve the quality of the proteins in our dataset and to avoid introducing proteins which were too large to process in a reasonable amount of time. Our testing dataset was composed of the target proteins in the CASP11 (The 11th Community Wide Experiment on the Critical Assessment of Techniques for Protein Structure Prediction) [21] dataset and was used to perform the final filtering on the training dataset. We used Clustal Omega [22] to filter homologous sequences from the training dataset if they had 25% or greater sequence similarity to any of the CASP11 proteins. Our final training dataset was composed of 1995 proteins after this filtering process. Instructions to reproduce this dataset can be found on the website for our RFcon web server.

### Prediction programs

Our feature generation process began with taking the sequences of our training proteins and using them as input for three prediction programs. We used PSIPRED version 3.5 [23] to predict secondary structure and ACCpro version 5.1 (a member of the SCRATCH 1.0 package) [24] to predict solvent accessibility. PSIPRED’s predictions are given for each residue in the sequence and are encoded in our examples as three probability values representing the likelihood of that residue being within either a β-sheet, α-helix, or a coil. ACCpro’s predictions are encoded for each residue in our examples with a binary number that represents whether the residue is solvent accessible or not [25].

DCA_cpp is our C++ implementation of the direct coupling analysis methods described by Morcos et al. [26] and was tested both as a part of the training data and as an independent prediction method in our evaluations. HHblits [27] was used to generate a multiple sequence alignment (MSA) against the Hidden Markov Model (HMM) database of “uniprot20_2015_06”. We used this MSA to obtain a sequence profile of the input protein sequence and a contact map with predictions scored by confidence value. Here the only input for DCA_cpp was the amino acid sequence of the proteins in our datasets.

### Feature generation process

We developed a python script that parsed the structure file of each protein in our datasets and combined the output of the previously mentioned prediction programs to generate the features that were used to train and test our machine learning models. Each example in this set of features represented the interaction between a pair of residues centered within two sliding windows that each contained 11 residues. For the models trained with binary classification, the target value (class label) of each example was set to positive (in contact) if the three dimensional Euclidean distance between the coordinates of the two residues’ α-carbons was less than or equal to 8 Å and set to negative (not in contact) otherwise. For the models trained using regression, the target value *t* was defined by the following equation where *d* is the Euclidean distance between the α-carbon atoms belonging to the residues at the center of each window, and *c* is a constant (set to 3 in our calculations).

4 $$ t=\frac{1}{1+{\left(\frac{\mathrm{d}}{\mathrm{c}}\right)}^2} $$

**Fig. 2:**
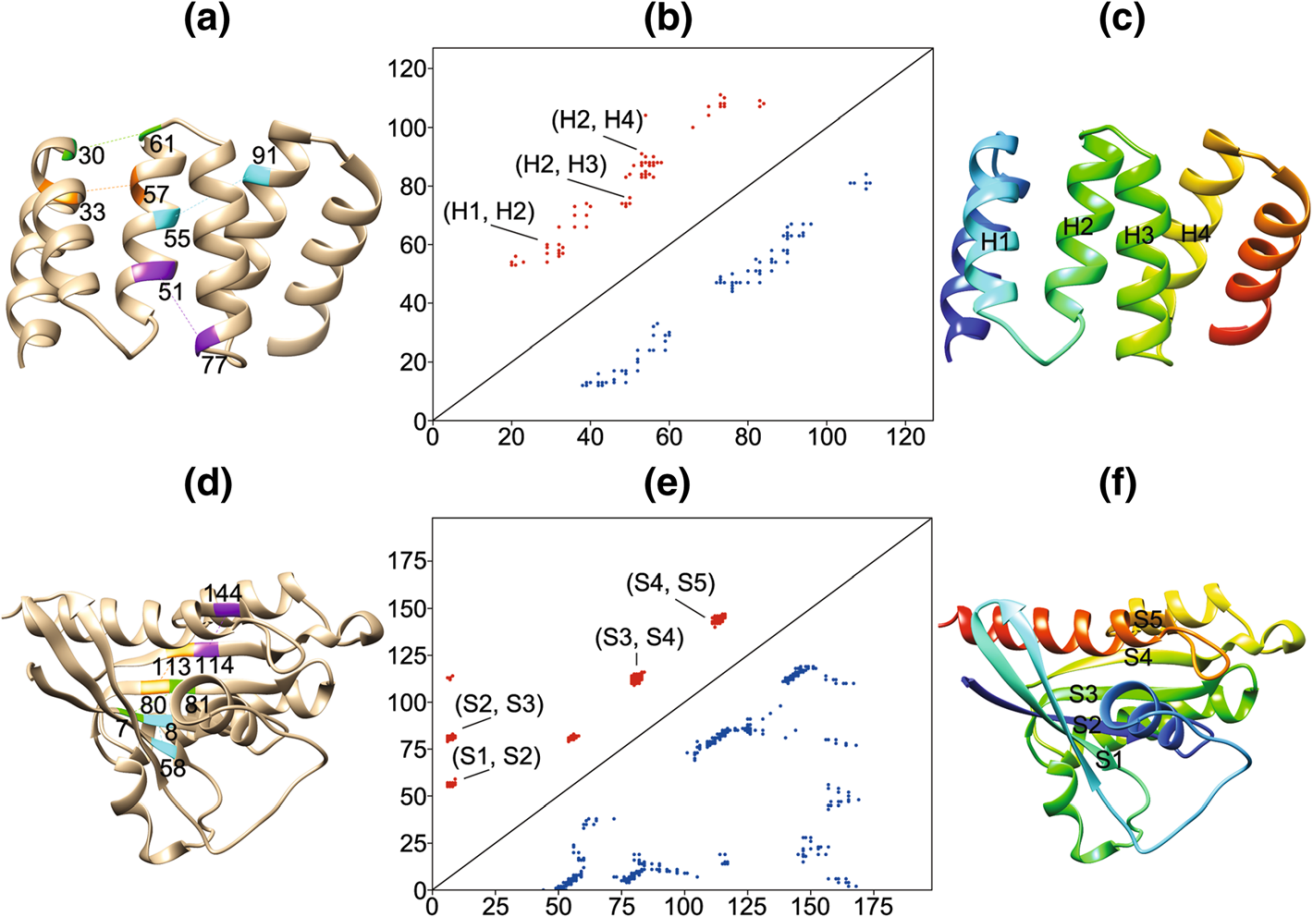
An example of the two window system. **a** “Empty” residue positions that are used because, in this configuration, the leftmost window extends beyond the range of the residues in the protein. **b** The first window (window “A” or the “left” window) centered at the first residue in the sequence. **c** The 50 residues between the end and beginning of the two windows. **d** The second window (window “B” or the “right” window) centered at residue 62 (in this case)

The action of sliding the windows along the sequence of the protein refers to the process for generating the feature data for each pair of residues used in the examples. During the parsing of each protein, the centers of the two windows were held at a minimum distance of 6 residues apart in the sequence at all times while sliding down the sequence of the protein. Figure 2 depicts the residues involved in one possible example with the first window (Fig. 2b) centered on the first residue of the protein and the second window (Fig. 2d) centered on the 62nd residue in the protein. Since the size of the window is static during feature generation, the first window (Fig. 2b) in this example extends past the boundary of the sequence and must be partially filled with “empty residues” (Fig. 2a). We encode these residues with the same number of features contained in regular residues. However, all of these values are set to 0 except for one feature (set to 1) to indicate that the residue is not part of the sequence. Regardless of the actual difference between the positions of the two window centers, 50 “intermediate” residues are always included even if duplicates are used (Fig. 2c). The sequence position of each residue *a*_*n*_ in this set is defined by the following formula where *n* is an integer from 1 to 50, *r* is the sequence position of the residue in the first window center, and *s* is a scale factor calculated by dividing the total number of residues between the two window centers by 50.

5 $$ {\mathrm{a}}_{\mathrm{n}}=\left\lfloor \mathrm{r}+\mathrm{sn}\right\rfloor $$

### Description of features

Each of the 72 residues included in every example was represented by a set of 32 features composed of various types of data. Five features described by Atchley et al. [28] were used to account for various physiochemical and biological properties of the amino acids. The sequence profile generated by DCA_cpp was encoded with 20 features. Both the Atchley features and these sequence profile features provide quantitative information that can describe the residue and its importance in the sequence more accurately. The remaining information used to describe each residue was represented with three features that encode PSIPRED’s secondary structure prediction, one feature that encodes ACCpro’s solvent accessibility prediction, two features that encode whether the residue is within the boundaries of one of the two windows or not, and one feature that encodes whether or not the residue is an “empty residue”. An empty residue is simply a place holder residue that is described by our feature generation script with a value of zero for every feature. This occurs when a residue needs to be encoded within a window that extends past the boundary of the protein. All of these values mentioned up until this point accounted for 2304 features in each example after picking up every combination of residues between the two sliding windows as described in the previous section. Then, 122 features were added to represent DCA_cpp’s contact prediction probabilities between the center of the first window and every residue until the center of the second window. Thus, the total number of local features which describe each data point is 2426.

In addition to these local features, 63 global features that described aspects of the protein as a whole were added to each example. Here, global featues are used to describe the protein as a whole rather than only one isolated example. For each example in the training data corresponding to one target protein, the global features remain constant. First, 20 features were used to encode the amino acid composition of the protein. These features represent the frequency of occurrence in the sequence for each amino acid. The next 40 features encode the pseudo amino acid composition of the protein as described by Chou [29]. The final three features in this category encode the exposed secondary structure composition. These features may assist in contact prediction by helping the model directly consider the shape of the protein at points of possible contact. These values represent the percentage of PSIPRED secondary structure predictions that were predicted to be exposed by ACCpro. In total, each example pair of residues is described with 2489 features. Additional details about these features can be found in the supplementary information (see Additional file 1).

Repeating this feature generation process for every pair of residues in the set of training proteins produced a dataset of examples that was then split into three groups based on the sequence separation (the difference of the numeric position between the residues in the two window centers) of the residue pairs in each group. The examples in these groups were organized with the separation range of 6–11 residues placed into the “sep6” group (199,536 examples), the separation range of 12–23 residues placed into the “sep12” group (245,914 examples), and the separation range of 24 residues and greater placed into the “sep24” group (743,600 examples). Since the number of negative examples gained during parsing the proteins was much greater than the number of positive examples, we retained all positive examples from each target protein but randomly sampled an equal number of negative examples.

### Feature selection

The large amount of features in our original training datasets required a very large amount of storage space and caused training to run very slowly with the machine learning methods we tested. Therefore, we used the randomForest [30] library in R to train three RF models on three subsets of 10,000 randomly selected examples (maintaining the balance of positive and negative examples and sequence separation categories) from the sep6, sep12, and sep24 datasets. These models were trained with the default “mtry” parameter, 1500 trees, and a “nodesize” of 10. The top 100 features with the highest “mean decrease Gini” values were then selected and three new datasets were created using only these 100 features from the full set of examples. This feature selection process was used to generate the features for all of our models described in the results section except for the “rf_full” models which used the full feature set. We also used this same procedure to select features based on the “mean decrease accuracy” metric. These features were used to train a different set of models. More details about the difference between these two sets of selected features can be found in the supplementary information (see Additional file 1).

### Model training and optimization

We trained our SVM models (svm) by using the SVM_light implementation [31] of the SVM algorithm with our three feature selected training datasets. Our SDA models (sda_balanced, sda_unbalanced, and sda_Ensemble) were generated with a Theano implementation of the SDA algorithm that we have utilized in previous studies [32–34]. We trained our random forest models (rf_full and rf_selected) using the randomForest library in R [30]. The training process for all of these models was guided by observing the cross-validation accuracy during training and choosing new parameters until no further improvement could be made. Here, we describe the training process used to determine the final parameters and architecture for the models used in each method. In order to more accurately compare the differences in performance among our own methods, all of our final models were optimized by tuning various hyperparameters to achieve high performance on our training dataset. The effect of tuning these various parameters was closely monitored and validated using standard cross-validation techniques for every method except for random forest, which uses out-of-bag error estimation to achieve similar optimization results.

### Support vector machine (svm)

Our final three SVM models were created by running SVM_light in classification mode using the linear kernel with the default parameters. Five-fold cross-validation was used to test the effects of different training parameters on the training dataset discussed in the methods section. We removed all of the examples in each training fold that were from proteins present in that training fold’s respective test fold and also maintained the equal class balance of positive and negative contact examples in all of the folds. For optimization purposes, we experimented with many of the different kernels available in SVM_light. More information about tuning our SVM models can be found in the supplementary information (see Additional file 1) and the results of an optimization evaluation can be found in the supplementary data (see Additional file 2).

### Stacked Denoising autoencoder (sda_balanced, sda_unbalanced, sda_Ensemble)

All of our final SDA-based models were trained with four-fold cross-validation by first splitting each of the three training datasets (the same as we used in the SVM training procedure) into two equal halves and removing duplicate proteins. We reserved one of the halves for training the final ensemble and then used the other half for training the individual SDA models. We prepared the data for the “sda_balanced” models by splitting the reserved model training data into two subsets of 25 and 75% of the reserved examples. These subsets were each split in half and duplicate proteins were removed. The final four folds were made by combining these subsets using each just once for pretraining, training, validation, and testing by the SDA algorithm. We used these folds to perform cross-validation and optimize our models. More information about the various models that we tuned can be found in the supplementary information (see Additional file 1).

The final three “sda_balanced” models were sep6 (one hidden layer with 33 units), sep12 (10 hidden layers with 30 units each), and sep24 (one hidden layer with 33 units). The “sda_unbalanced” models were created by taking the four folds used for training the “sda_balanced” models and randomly downsampling the positives examples to a ratio of one positive example to every five negative examples. The final three “sda_unbalanced” models were sep6 (one hidden layer with 66 units), sep12 (five hidden layers each with 80 units), and sep24 (one hidden layer with 80 units and a second hidden layer with 40 units). All of the hidden layers in our SDA models shared a common corruption value of 0.1.

The “sda_Ensemble” models were trained by selecting a group of several of the “sda_unbalanced” models and combining them with an SVM model. This was done by selecting some of the highest performing models and using their predictions for the reserved data as the only input for training the final ensemble SVM models with linear regression. After verifying performance with five-fold cross-validation, the final ensemble models were sep6 (10 sda_unbalanced models, SVM -c parameter = 0.01), sep12 (10 sda_unbalanced models, default SVM parameters), and sep24 (5 sda_unbalanced models, default SVM parameters).

### Random Forest (rf_full and rf_selected)

Our first three random forest models were trained on the datasets that had undergone feature selection. After trying various parameters and observing the effect on the OOB (Out Of Bag) estimate of the error on the training data, we used 1501 trees, a nodesize of 10, and the default value of mtry for all three of these “rf_selected” models. The last three models (“rf_full”) were trained on the full feature set before feature selection had been performed. The model trained on the full sep6 dataset used the same training parameters as the previously feature selected models. As a result of memory and computational time limitations, we chose different parameters for the sep12 and sep24 models. We chose to use 551 trees for these two models since the OOB error on the training set stopped noticeably decreasing at this point. The nodesize parameter was set to 10, and “mtry” was left at the default value.

### Additional models

In order to more closely examine our features and their importance, we also trained all of the previously mentioned models using mean decrease accuracy feature selection rather than mean decrease gini. We performed this feature selection using the same process and random forest models that generated the mean decrease gini featues. This allowed us to compare two sets of selected features for each sequence separation category and observe the effects of training models using them. In addition to this, we trained a selection of the original feature selection models (mean decrease gini) using only 50% of the features.

## Additional files

Additional file 1: Supplementary Information. This document provides more details regarding the feature generation and feature selection process used in this study. It also discusses our model optimization process and explicitly describes each type of feature and how they are combined to create a single example (data point). (DOCX 171 kb)

Additional file 2: Supplementary Data (model name and evaluation criteria). This file includes the training evaluation results for our svm_sep6_balanced model. Specifically, it shows the performance of each candidate model on the training dataset. A more detailed description of this data and the hyperparameters associated with each model can be found in the supplementary information (see Additional file 1). (XLSX 15 kb)

## Abbreviations

acc: Accuracy
CASP11: The 11th Community Wide Experiment on the Critical Assessment of Techniques for Protein Structure Prediction
CNN: Convolutional Neural Network
cov: Coverage
DCA: Direct coupling analysis
DNs: Deep networks
HMM: Hidden Markov Model
MILP: Mixed integer linear programming
MSA: Multiple sequence alignment
RBMs: Restricted Boltzmann machines
RF: Random forest
SDA: Stacked denoising autoencoders
SVMs: Support vector machines
TM: Transmembrane
