# Supplementary material for: Predicting protein residue-residue contacts using random forests and deep networks
Source: BMC Bioinformatics. 2019 Mar 14;20(Suppl 2):100. doi: 10.1186/s12859-019-2627-6 (PMC6419322; doi:10.1186/s12859-019-2627-6)
Supplement: Supplementary file 1 — Supplementary Information. This document provides more details regarding the feature generation and feature selection process used in this study. It also discusses our model optimization process and explicitly describes each type of feature and how they are combined to create a single example (data point). (DOCX 171 kb) [file 12859_2019_2627_MOESM1_ESM.docx]

**Supplementary Information for: Predicting protein residue-residue contacts using random forests and deep networks**

Joseph Luttrell IV^1^, Tong Liu^2^, Chaoyang Zhang^1^, Zheng Wang^*, 2^

* Corresponding author: Zheng Wang, zheng.wang@miami.edu

1. School of Computing Sciences and Computer Engineering, University of Southern Mississippi, 118 College Drive #5106, Hattiesburg, MS 39406, USA

2. Department of Computer Science, University of Miami, 1365 Memorial Drive, Coral Gables, FL 33124, USA

Author Emails:

JL: joseph.luttrell@usm.edu

TL: tong.liu@miami.edu

CZ: chaoyang.zhang@usm.edu

ZW: zheng.wang@miami.edu

**Detailed Description of Features**

Here, we list each type of feature used to train our machine learning models. Also, we will more concisely describe the feature composition of our data points. Each data point (example) in our datasets can be described by a set of 2,426 local features and 63 global features. However, there are only 10 types of features that describe an individual residue in our data. The large set of features which describes each of our data points is generated by the sliding window process outlined in the main manuscript under the feature generation process section.

The following list of components describes a single data point after the sliding window process has gathered the required 72 residues.

Part One: Each residue is composed of the following amounts of these 7 distinct types of features (which are described in detail under the Description of Features section in the main manuscript)

(3*ss + 1*sa + 1*wa + 1*wb + 1*ob + 20*prof + 5*atch) = 32 features

where

- ss = PSIPRED's secondary structure prediction (a set of three probabilities reflecting the likelihood of the secondary structure at each residue being either β-sheet, α-helix, or a coil).
- sa = solvent accessibility predictions generated by ACCpro (a binary feature that predicts whether the particular residue is accessible to the solvent or not).
- prof = sequence profile information frrom DCA_cpp (described in the main manuscript with more details in the provided reference).
- dca = dca contact probability values from DCA_cpp (described in the main manuscript with more details in the provided reference).
- wa = window center a (a single binary feature representing the left/first center of each sliding window pair).
- wb = window center b (a single binary feature representing the right/second center of each sliding window pair).
- atch = atchley values (A set of five factor solution scores describing the multidimensional patterns of attribute covariation within amino acids. These values reflect polarity, secondary structure, molecular volume, codon diversity, and electrostatic charge).

After the sliding window process is complete, as described in the Feature Generation section of the main manuscript, 72 of these residues have been obtained. At this point, 122 features which encode the dca contact map for each residue are added (a redundant collection of the contact probability map for 50 residues between the window centers + 11 in the opposite window combined with the same features in the opposite direction.)

Part Two: Global features are calculated for each target protein and added to the end of each example in that target. These examples are encoded globally. Therefore, they remain constant for each data point which describes a single target protein. Each set of global features is composed of the following 3 types of features, and they are described in more detail in the main feature description section of the main manuscript.

(20gaa + 40paa + 3gess) = 63 features

where

- gaa = global amino acid composition (20 features that describe the frequency of each residue in the target protein as a whole).
- paa = pseudo amino acid composition (described in the main manuscript)
- gess = global exposed secondary structure composition (3 features that encode the frequency of each type of secondary structure at residues that are exposed to the solvent. Can be either β-sheet, α-helix, or a coil).

Part Three: After each iteration of the sliding window mechanism described in the main manuscript, 2,426 features have been encoded for a single example. Then, the global features described in Part Two above are added. This brings the total feature count for each example (data point) to 2,489. This number represents a combination of the 10 types of features we have described. The values for these features are simply sampled from different areas of the protein.

**Model Optimization**

In order to more accurately compare the differences in performance among our own methods, all of our final models were optimized by tuning various hyperparameters to achieve high performance on our training dataset. The effect of tuning these various parameters was closely monitored and validated using standard cross-validation techniques for every method except for random forest, which uses out-of-bag error estimation to achieve similar optimization results.

**SVM**

We have tried training models using every kernel offered by SVM_light. For our final models, we chose the linear kernel for a number of reasons. On our dataset, running SVM_light in classification mode with the linear kernel gave relatively good out-of-the-box performance with only small adjustments to parameters such as C (the trade-off between training error and the margin). Any large adjustments quickly caused our models to overfit according to our measurements of accuracy on the training dataset. Therefore, our models were chosen to not only have a high accuracy on the training dataset, but also to have stable performance on all folds of the cross-validation. **Supplementary Table 1** illustrates this process by showing the parameters tried when training the final model used for svm_sep6_balanced. It also shows the plethora of other models from which it was chosen. Corresponding accuracy and coverage values for this evaluation can be found in the **Supplementary Data** [see Additional file 2].

**SDA**

For sda_balanced, sda_unbalanced, and the models which made up the sdaEns (sda ensemble) models, we experimented with a large number of hidden layer configurations, corruption levels, numbers of training/pretraining epochs, and fine tuning learning rates. Our process for picking the final models was identical to the process described above used to select the final SVM models. For example, when training the sda_unbalanced model for sep6, we tried up to 5 hidden layers. We tried a different number of units in each hidden layer ranging from 10 to 800. We also tried corruption levels for each hidden layer ranging from 0.1 to 0.7. This describes the amount of corruption applied to the input during training of the sda model. The goal of training the sda is to be able to successfully perform reconstruction of the input regardless of the corruption. The effects of changing all of these parameters were noted and applied to training our other sda models as guidance. For the sda_unbalanced models of the sep6 category alone, 400 models were trained and evaluated (a set of 100 unique parameter and architecture combinations).

**RF**

For random forest models, we found that much less optimization was typically needed to achieve acceptable results out-of-the-box. However, we did try different nodesize values ranging from 2 to 10, and different values of mtry ranging from 10 to 95. Also, we tried different numbers of trees ranging from 100 to 1500 in increments of 50 and saw improvement from reaching 1500 trees. The act of selecting features according to mean decrease in Gini is essentially based on measuring the average gain of purity by splits of a given variable. In other words, a variable will score higher in this test if it is more useful at discriminating between classes. This means that it will more effectively produce single class nodes from nodes with mixed labels. The 'meanDecreaseAccuracy' metric, on the other hand, focuses on excluding single variables during training and measuring the resulting decrease in the accuracy of the random forest. For both metrics, the higher scoring variables are selected.

In **Supplementary Figure 1** we give an example of the results of this process and show our top 20 features selected according to the 'meanDecreaseGini' metric for the sep6 dataset. Also, in **Supplementary Figure 2** we show a different set of top 20 features selected with the 'meanDecreaseAccuracy' metric on the sep 6 dataset. The two types of features which show up here ('ss1' and 'pro7') are secondary structure and sequence profile features respectively. More details about each feature type and the generation of the full feature set can be found in the main manuscript as well as the Detailed Description of Features section here.

**Feature Selection**

As described in the manuscript, we used the randomForest package in R to perform two types of feature selection on our data. First, we trained models with the top 100 features selected with the metric 'meanDecreaseGini'. Then, we trained models using the same parameters but with 'meanDecreaseGini' as the feature selection metric instead.

**Supplementary Tables**

Supplementary Table 1 SVM model c parameter optimization per model family. For each model family (Xmodel_#), a set of 5 models was trained using cross-validation. The names of each family correspond to the accuracy values shown in Supplementary Figure 1.

| **model name** | **C parameter value** |
| --- | --- |
| Xmodel_0 | default |
| Xmodel_1 | 10 |
| Xmodel_2 | 5 |
| Xmodel_3 | 0.1 |
| Xmodel_4 | 0.01 |
| Xmodel_5 | 0.001 |
| Xmodel_6 | 0.0001 |
| Xmodel_7 | 0.00001 |
| Xmodel_8 | 0.000001 |
| Xmodel_9 | 0.0000001 |
| Xmodel_10 | 15 |
| Xmodel_11 | 30 |
| Xmodel_12 | 100 |
| Xmodel_13 | 150 |
| Xmodel_14 | 200 |
| Xmodel_15 | 210 |
| Xmodel_16 | 250 |
| Xmodel_17 | 300 |
| Xmodel_18 | 350 |
| Xmodel_19 | 400 |
| Xmodel_20 | 35 |
| Xmodel_21 | 45 |
| Xmodel_22 | 65 |
| Xmodel_23 | 85 |
| Xmodel_24 | 90 |
| Xmodel_25 | 48 |
| Xmodel_26 | 52 |
| Xmodel_27 | 55 |
| Xmodel_28 | 60 |
| Xmodel_29 | 63 |

**Supplementary Figures**

**
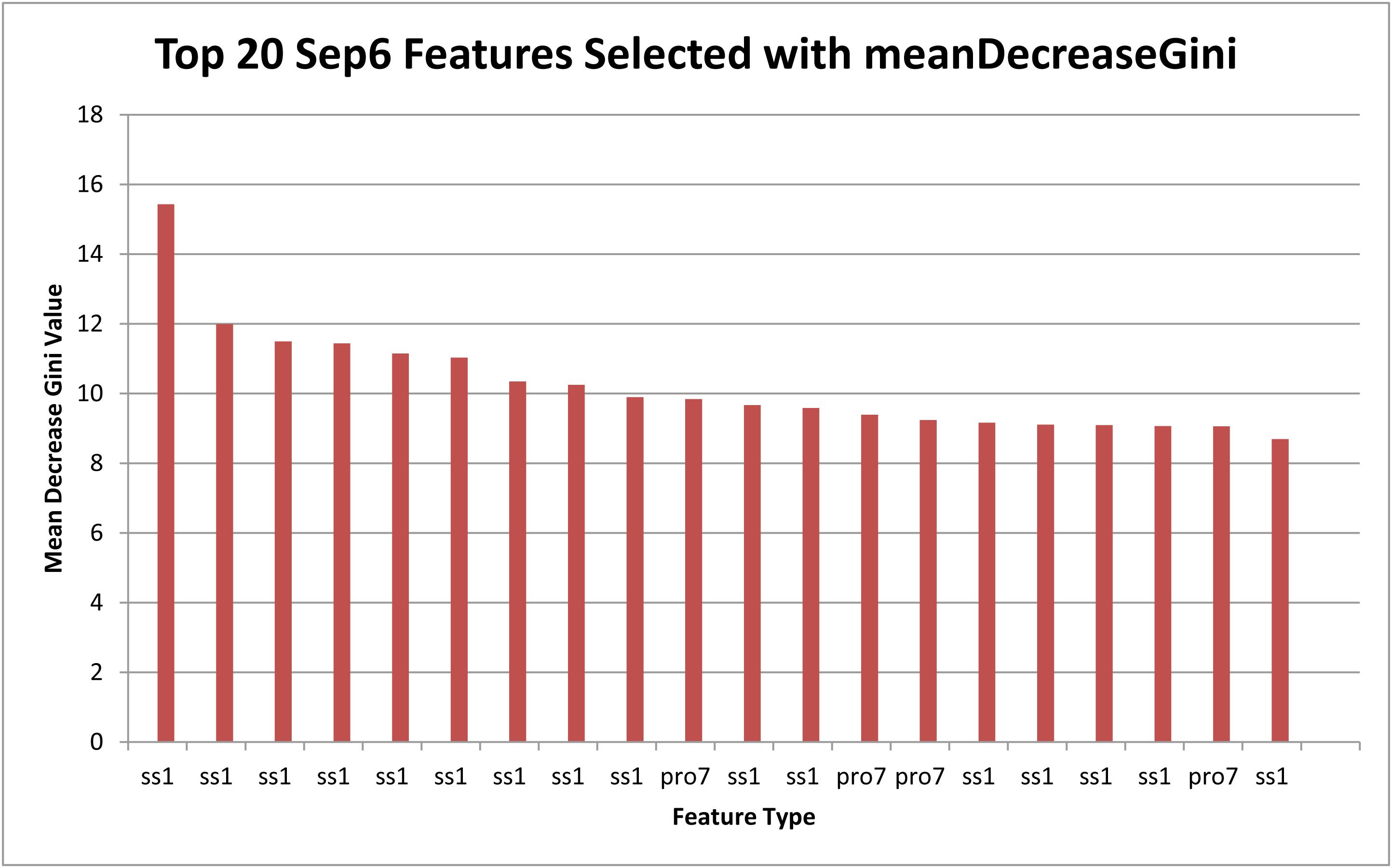
**

**Supplementary Figure 1** Feature type vs. importance ranked with meanDecreaseGini.


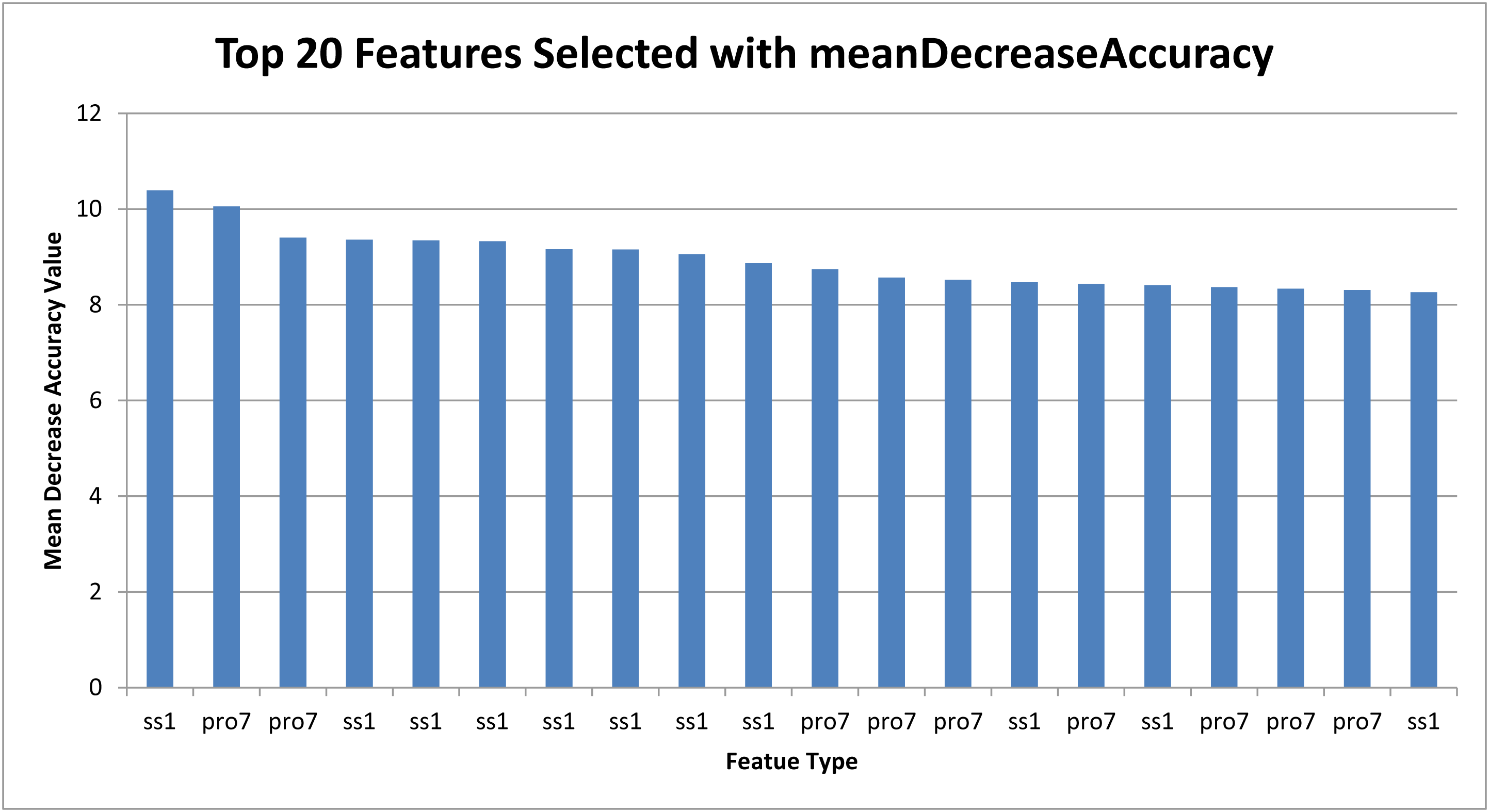


**Supplementary Figure 2** Feature type vs. importance ranked with meanDecreaseAccuracy
